# Chemical Characterization of Fruit Wine Made from Oblačinska Sour Cherry

**DOI:** 10.1155/2014/454797

**Published:** 2014-06-29

**Authors:** Milica Pantelić, Dragana Dabić, Saša Matijašević, Sonja Davidović, Biljana Dojčinović, Dušanka Milojković-Opsenica, Živoslav Tešić, Maja Natić

**Affiliations:** ^1^Faculty of Chemistry, University of Belgrade, P.O. Box 51, 11158 Belgrade, Serbia; ^2^Innovation Center, Faculty of Chemistry Ltd, University of Belgrade, 11158 Belgrade, Serbia; ^3^Faculty of Agriculture, University of Belgrade, Nemanjina 6, Zemun, 11080 Belgrade, Serbia; ^4^Centre of Chemistry, Institute of Chemistry, Technology and Metallurgy, University of Belgrade, 11000 Belgrade, Serbia

## Abstract

This paper was aimed at characterizing the wine obtained from Oblačinska, a native sour cherry cultivar. To the best of our knowledge, this is the first paper with the most comprehensive information on chemical characterization of Oblačinska sour cherry wine. The chemical composition was characterized by hyphenated chromatographic methods and traditional analytical techniques. A total of 24 compounds were quantified using the available standards and another 22 phenolic compounds were identified based on the accurate mass spectrographic search. Values of total phenolics content, total anthocyanin content, and radical scavenging activity for cherry wine sample were 1.938 mg gallic acid eqv L^−1^, 0.113 mg cyanidin-3-glucoside L^−1^, and 34.56%, respectively. In general, cherry wine polyphenolics in terms of nonanthocyanins and anthocyanins were shown to be distinctive when compared to grape wines. Naringenin and apigenin were characteristic only for cherry wine, and seven anthocyanins were distinctive for cherry wine.

## 1. Introduction

Oblačinska sour cherry is a native cultivar. High fruit quality, self-fertility, and high demand in domestic and foreign fruit markets makes the Oblačinska sour cherry the most planted cultivar in Serbian commercial orchards [1]. The fruit is of “morello” type, small to medium in size, with dark red and thin skin. The flesh is red, medium-firm, juicy, quite sour aromatic and of high quality [2]. Serbia is one of the leading world producers of this commercially very important fruit species [3].

Generally, cherries are important sources of polyphenols such as anthocyanins, flavan-3-ols and flavonols [4]. Also, they are characterized by a high phenolic acid content, especially hydroxycinamic and hydroxybenzoic acid derivates [5, 6]. Wide ranges of phenolic secondary metabolites are associated with antioxidizing capacity, nutritional, and therapeutic value of cherries [7]. In sour cherries anthocyanins are considered as major group of phenolic compounds [8]. This class of compounds was shown to prevent cardiovascular diseases and to possess strong anti-inflammatory and anticarcinogenic activity [9, 10]. In the literature, several papers reported cyanidin-3-glucoside, cyanidin-3-rutinoside, cyanidin-3-glucosylrutinoside, cyanidin-3-sophoroside, pelargonidin-3-glucoside, peonidin-3-rutinoside and cyanidin-3-arabinosylrutinoside as main anthocyanins in sour and sweet cherry fruits [5, 10, 11].

Sour cherries are characterized with sufficient acid level and are preferred for winemaking, using very similar procedure to the grape wine making process [4]. Continuing our research in the field of chemical characterization of fruits and wines from Serbia we found interesting to screen phytochemical composition of wine obtained from Oblačinska sour cherry cultivar. Previously we have reported on chemical composition of peach wine produced from Redhaven cultivar belonging to same genus (*Prunus*) as cherry [12]. Special attention was given to composition of polyphenols, an important group of phytochemicals which are reported to be associated with antioxidant activity and have several health effects. In this work, two methods based on hyphenated techniques, which combine chromatographic and spectral methods, were used for target search and establishing polyphenolic profiles. Ultra High Performance Liquid chromatography (UHPLC) coupled with hybrid mass spectrometer which combines the Linear Trap Quadrupole (LTQ) and OrbiTrap mass analyzer was used to identify phenolics. Quantification of phenolics was done using UHPLC coupled with a diode array detector (DAD) and connected to a triple-quadrupole mass spectrometer. Also, the overall antioxidant activities, total phenolic content (TPC), total anthocyanin content (TAC), and mineral content were determined. In order to gain insight into the value of the polyphenolic composition and nutritional quality of cherry wine, red wines produced from three different grape cultivars were also analyzed. The results obtained for cherry wine were compared with the results of selected red wines.

## 2. Materials and Methods

### 2.1. Materials and Chemicals

2,2-Diphenyl-1-picrylhydrazyl^*∙*^ (DPPH^*∙*^), standards of gentisic acid, (+)-catechin, (+)-gallocatechin, aesculin, (−)-epigallocatechin,* p*-hydroxybenzoic acid, protocatechuic acid, (−)-epicatechin, (−)-gallocatechin gallate, rutin, ellagic acid, naringin, (−)-epigallocatechin gallate, myrcetin, quercetin, naringenin, luteolin, chrysin, pinocembrin, galangin,* trans*-resveratrol, apigenin, and hesperetin were purchased from Fluka AG (Buch, Switzerland). The other compounds namely* cis,trans*-abscisic acid, gallic acid, chlorogenic acid, caffeic acid,* p*-coumaric acid, nitric acid, and hydrogen peroxide were supplied by Sigma Aldrich (Steinheim, Germany). Methanol (HPLC grade), acetonitrile, and formic acid (both of them MS grade), sodium carbonate, potassium chloride, acetic acid, hydrochloric acid, sodium acetate, ethyl acetate, and Folin-Ciocalteu reagent were purchased from Merck (Darmstadt, Germany). Ultrapure water (TKA Germany MicroPure water purification system, 0.055 *μ*S cm^−1^) was used to prepare standard solutions and dilutions. All other reagents were of analytical grade. Syringe filters (13 mm, PTFE membrane 0.45 *μ*m) were purchased from Supelco (Bellefonte, PA, USA). The SPE cartridges Strata C18-E (500 mg/3 mL) were obtained from Phenomenex (Torrance, CA, USA).

### 2.2. Preparation of Standard Solutions

A 1000 mg L^−1^ stock solution of a mixture of polyphenolics and* cis,trans*-abscisic acid were prepared in methanol. Dilution of the stock solution with methanol yielded the working solution at concentrations of 0.025, 0.050, 0.100, 0.250, 0.500, 0.750, and 1.000 mg L^−1^. Calibration curves were obtained by plotting the peak areas of the compounds identified against the concentration of the standard solution.

### 2.3. Wine Samples

The tests were performed on one sample of cherry wine-East Serbia (SCW) and five samples of grape wines (Vranac-East Serbia (W1), Cabernet Sauvignon-Central Serbia (W2), Cabernet Sauvignon-East Serbia (W3), Cabernet Sauvignon-North Serbia (W4) and Frankovka-North Serbia (W5)).

Cherry wine was made using technological procedure for red wine production, which includes sour cherry mushing (disintegration), mushed sour cherry maceration, alcoholic fermentation and removal of skins, seeds, and stalks from the wine. Harvesting of Oblačinska sour cherry was done at its commercial ripening stage, when fruits showed the highest values of soluble solids (which were proved by hand refractometer) having dry stem scar. Also, fruits showed uniform color across at least 90% of the fruit skin with the characteristic traits of a given cultivar. Harvest was followed by mushing, disintegration of the fruit and disposal of husks and stalks in fermentation containers. Temperature-controlled fermentors were used for fermentation (18–20°C) with appropriate enzymes facilitating separation of colored and aromatic matters during the maceration.

### 2.4. Samples Preparation

All wine samples were filtered through a 0.45 *μ*m PTFE filters before analyzing. For all tests, samples were appropriately diluted. Solid phase extraction (SPE) was used for separation of anthocyanin and non-anthocyanin fraction. First, C18 cartridges were preconditioned by passing through 10 mL ethyl acetate, 10 mL methanol, and 10 mL of 0.1 mol/dm^3^ aqueous HCl, sequentially. Then, the amounts of 0.5 mL of extracts were applied. Cartridges were washed with 10 mL of 0.1 mol/dm^3^ aqueous HCl in order to remove sugars, acids, and other water-soluble compounds. Cartridges were dried by allowing a current of nitrogen gas to pass through, for 5 minutes, and after that, rinsed with 5 mL ethyl acetate in order to collect non-anthocyanins fraction. The adsorbed anthocyanins were eluted from the cartridges with 1 mL acidic methanol.

### 2.5. Determination of TPC

The concentration of total soluble phenolics was determined spectrophotometrically on a UV-Vis spectrophotometer (GBC UV-Visible Cintra 6) and was measured using Folin-Ciocalteu method, with some modifications [13]. This reagent oxidizes phenols present in the solution. An aliquot (0.1 mL) of appropriately diluted samples and a standard solution of gallic acid were mixed with 6 mL deionized water and 0.5 mL of Folin-Ciocalteu reagent. After 6 min, 1.5 mL of 2 mol/dm^3^ sodium carbonate was added with mixing. After incubation for 60 min at 40°C, the absorbance was measured at 765 nm and it was proportional to the total quantity of phenolic compounds. Gallic acid was used as a standard. TPC was expressed as the g gallic acid equivalent (GAE) per L of sample.

### 2.6. Determination of TAC

In an acid environment there is a balance between the two forms of anthocyanins, colored and the colorless, depending on the pH. Total anthocyanin contents were determined using the pH-differential method, by measuring the absorbance of the sample at pH = 1 (KCl, 0.025 mol L^−1^) and pH = 4.5 (NaOAc/HOAc, 0.4 mol L^−1^) [14]. Measurements were performed at two wavelengths, at 520 nm and 700 nm, against a blank cell filled with distilled water. Anthocyanin concentrations were calculated and expressed as g malvidin-3-glucoside (mal-3-glu) equivalents per 1 L sample. The total absorbance of the extract was determined by the formula:

(1) $$\begin{array}{c} A=\left[{\left(A_{520}-A_{700}\right)}_{\text{pH}1}-{\left(A_{520}-A_{700}\right)}_{\text{pH}4,5}\right]. \end{array}$$

The content of total anthocyanins (TAC) was determined through the following formula:

(2) $$\begin{array}{c} \text{TAC}\;\left(\text{mg}\;{\text{L}}^{-1}\right)=\frac{\left(A\cdot \text{MW}\cdot \text{DF}\cdot 1000\right)}{\left(\varepsilon \cdot 1\right)}, \end{array}$$

where *A* is absorbance, MW is molecular weight (MW = 493.2 g mol^−1^ for malvidin-3-glucoside), DF is dilution factor, 1 is cuvette path length in cm, *ε* is molar absorptivity (*ε* = 28000 Lmol^−1^cm^−1^ for malvidin-3-glucoside).

### 2.7. Determination of the RSA

Radical scavenging activity, the ability to scavenge DPPH free radicals, was determined spectrophotometrically [15]. Amounts of 0.1 mL of appropriately diluted samples solutions were mixed with 4 mL of methanol DPPH radical solution. The mixtures were placed in the dark, at room temperature. After an incubation period of 30 min, the absorbance was measured at 515 nm to determine the concentration of remaining DPPH^*∙*^. Relative antioxidant activity was calculated using the following formula:

(3) $$\begin{array}{c} \text{RSA}\left(\%\right)=\left(\frac{\left(A_{\text{DPPH}}-A_{\text{sample}}\right)}{A_{\text{DPPH}}}\right)\cdot 100, \end{array}$$

*A*_DPPH_ is the absorbance of methanol solution of DPPH radical, *A*_sample_ is the absorbance in the presence of wine. Measurements were performed in triplicate and the results were expressed as mean values.

### 2.8. Identification of Polyphenolic Compounds Using LC-MS/MS Analysis

All experiments were performed using a Thermo Fisher Scientific instruments.

Separation, determination and quantification of compounds of interest in each sample were performed using Dionex Ultimate 3000 UHPLC system equipped with a diode array detector (DAD) and connected to a triple-quadrupole mass spectrometer. Elution was performed at 40°C on Syncronis C18 column (100 × 2.1 mm, 1.7 *μ*m particle size, Thermo Fisher Scientific, USA). The mobile phase consisted of (A) water + 0.2% formic acid, and (B) acetonitrile, which were applied in the following gradient elution: 0.0–2.0 min 5% B, 2.0–12.0 min from 5% to 95% (B), 12.0-12.1 min from 95% to 5% (B), then 5% (B) for 3 min. The flow rate was set to 0.4 mL min^−1^ and the detection wavelength to 280 nm. The injection volume was 5 *μ*L.

Quantitative analysis of each sample was performed on a TSQ Quantum Access Max triplequadrupole mass spectrometer (Thermo Fisher Scientific, Basel, Switzerland), equipped with heated electrospray ionization (HESI) source. The ion source settings were set as follows: spray voltage was 4000 V; sheet gas pressure 40 arbitrary units, sweep pressure 0 arbitrary units, and auxiliary gas pressure was 8 arbitrary units; capillary temperature at 300°C; skimmer offset was 0 V. Mass spectrometry data were acquired in a negative mode. Collision-induced fragmentation experiments were performed using argon as the collision gas, and collision energy was set to 30 eV. Selected reaction monitoring (SRM) experiment for quantitative analysis was performed using two MS^2^ fragments for each compound, which were previously defined as dominant in PIS (precursor ion scan) experiments.

UHPLC ± MS/MS Orbitrap analysis of polyphenolic compounds were performed using a Thermo Scientific liquid chromatography system consisting of a quaternary Accela 600 pump and Accela Autosampler, connected to a linear ion trap-orbitrap hybrid mass spectrometer with heated-electrospray ionization probe (HESI-II, ThermoFisher Scientific, Bremen, Germany). Phenolics were identified and quantified in wine samples according to the corresponding spectral characteristics: mass spectra, exact mass, characteristic fragmentation, and characteristic retention time. Xcalibur software (version 2.1) was used for instrument control, data acquisition and data analysis. Internet database of accurate mass spectrometry data, ChemSpider (http://www.chemspider.com/), was used as a reference library to identify compounds of interest. MS spectra were acquired by full range acquisition covering 100–1000* mz* ^−1^. For fragmentation study, a data dependant scan was performed by deploying the collision-induced dissociation (CID). The normalized collision energy of the collision-induced dissociation (CID) cell was set at 35 eV.

Separation of polyphenolics was performed on a Hypersil gold C18 (100 × 2.1 mm, 1.9 *μ*m) from Thermo Fisher Scientific. The mobile phase consisted of (A) water + 0.1% formic acid and (B) acetonitrile + 0.1% formic acid. A linear gradient program at a flow rate of 0.300 mL min^−1^ was used: 0.0–1.0 min 5% B, 1.0–9.9 min from 5% to 95% (B), 9.9-10 min from 95% to 5% (B), then 5% (B) for 3 min. The injection volume was 5 *μ*L. The mass spectrometer was operated in negative ionization mode. HESI-source parameters were as follows: source voltage 3 kV, capillary voltage −20 V, tube lens voltage −150 V, capillary temperature 275°C, sheath and auxiliary gas flow (N_2_) 30 and 8 (arbitrary units).

Separation of anthocyanins was performed on a Hypersil gold C18 (100 × 2.1 mm, 1.9 *μ*m) from Thermo Fisher Scientific. The mobile phase consisted of (A) water + 1% formic acid and (B) acetonitrile. A linear gradient program at a flow rate of 0.300 mL min^−1^ was used: 0.0–2.0 min 5% B, 2.0–12.0 min from 5% to 95% (B), 12.0–12.2 min from 95% to 5% (B), then 5% (B) for 3 min. The injection volume was 5 *μ*L. The mass spectrometer was operated in positive ionization mode. HESI-source parameters were as follows: source voltage 5 kV, capillary voltage 40 V, tube lens voltage 125 V, capillary temperature 275°C, sheath and auxiliary gas flow (N_2_) 30 and 8 (arbitrary units).

### 2.9. Determination of Minerals

All wine samples were evaporated down to half their original volume by rotary evaporation under reduced pressure at 40°C and these solutions were prepared by microwave digestion using an Ethos 1 microwave system (Advanced Microwave Digestion System, Milestone, Italy). About 10 g of evaporated solutions, 5 mL of 65% HNO_3_ and 1 mL 30% H_2_O_2_ were mixed and transferred by pouring into the microwave digestion vessel. After effervescence subsided samples were cooled for five minutes, transferred into volumetric flasks, and diluted to 25 mL with deionized H_2_O. Blank was prepared in the same way. All analyses were performed on a Thermo Scientific iCAP 6500 Duo ICP (Thermo Fisher Scientific, Cambridge, UK).

## 3. Results and Discussion

### 3.1. Determination of TPC, TAC and RSA

Total phenolic contents, total anthocyanin contents, and antioxidant activity of six wine samples are listed in Table 1. Based on these results, it can be seen that the highest TPC value is presented in wine Cabernet Sauvignon-East Serbia (2.50 g GAE L^−1^ wine), followed by the Cabernet Sauvignon-Central Serbia (2.28 g GAE L^−1^ wine). The lowest TPC was recorded in Cabernet Sauvignon-North Serbia (1.19 g GAE L^−1^ wine). TPC value for cherry wine was 1.94 g GAE L^−1^ wine, which is in agreement with the data found in the literature [16]. The anthocyanin contents in studied wine samples were in the range from 0.08 g mal-3-glu L^−1^ wine (in Vranac) to 0.22 g mal-3-glu L^−1^ wine (Frankovka). All wine samples showed distinct radical-scavenging activities. The highest values were found for Cabernet Sauvignon-East Serbia (48.60%) and Cabernet Sauvignon-Central Serbia (46.41%) and the lowest for Cabernet Sauvignon-North Serbia (21.00%). The results obtained for the relative antioxidant activity were compared with the content of total phenolics and anthocyanins. The results show satisfactory correlation coefficient for TPC and RSA, indicating a statistically strong linear relationship (*r* = 0.990). General conclusion can be made regarding comparison of TPC, TAC and RSA values: in the case of cherry wine all the values are in the range of the results obtained for all investigated wines.

### 3.2. Quantitative and Qualitative Characterization of Wine Samples

**Figure 1:**
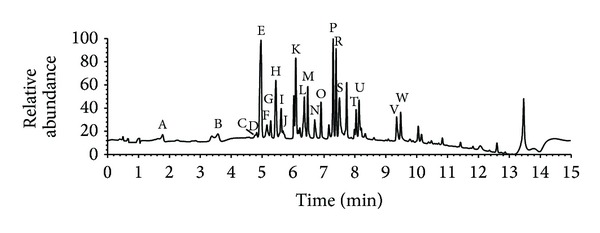
UV chromatogram of investigated standards at 280 nm: Gallic acid (A); Protocatechuic acid and (+)-Gallocatechin (B); Aesculin (C); (−)-Epigallocatechin (D);* p*-Hydroxybenzoic and Gentisic acid (E); Chlorogenic acid (F); (+)-Catechin (G); Caffeic acid (H); (−)-Epicatechin (I); (−)-Gallocatechin gallate (J); Rutin (K);* p*-Coumaric and Ellagic acid (L); Naringin (M); (−)-Epigallocatechin gallate (N); Myrcetin (O);* cis,trans*-Abscisic acid (P); Luteolin (R); Quercetin and* trans*-Resveratrol (S); Naringenin (T); Apigenin (U); Chrysin and Pinocembrin (V); Hesperetin and Galangin (W).

Great help in determining the polyphenolic profiles comes from the hyphenated techniques, which combine chromatographic and spectral methods. Here UHPLC-DAD MS/MS and UHPLC ± MS/MS Orbitrap analysis were utilized in order to obtain a comprehensive picture of individual flavonoids and phenolic acids, both in qualitative and quantitative sense. A total of 24 compounds were quantified using the available standards (Table 2). UV chromatogram of investigated standards at 280 nm is shown in Figure 1. The limits of detection and quantification, and the recoveries of the analytes were determined according to the method described by Gašić et al. [17]. Calibration curves revealed good linearity, with *R* ^2^ values exceeding 0.99 (peak areas vs. concentration). Recoveries determined for phenolic acids were 60% to 80%, while for flavonoids they were 80% to 120%. Additionally, 22 phenolic compounds were identified based on accurate mass search. In the absence of standards, the identification of the corresponding compound was based on the search for the [M–H]^−^ deprotonated molecule together with the interpretation of its fragmentations. Their mean expected retention times (*t*_*R*_), calculated mass, found mass, mean mass accuracy (ppm), and MS/MS fragments for each of the identified compound and their distribution in wines are summarized in Table 3.

Characteristic compounds are specified and major differences between cherry wine and grape wine samples are emphasized in the following discussion. Total of 7 phenolic acids were quantified in cherry wine using available standards (gallic acid, protocatechuic acid,* p*-hydroxybenzoic acid, gentisic acid, chlorogenic acid, caffeic acid, and* p*-coumaric acid). Two isomers of caffeoylquinic acids and syringic acid were found based on accurate mass search. Here, it is important to point out a higher content of protocatechuic acid, chlorogenic acid, caffeic acid, and* p*-coumaric acid in wine produced from sour cherry when compared to grape wine samples (see Table 2) which is in accordance with findings reported for the sour cherry fruit [18]. Total content of phenolic acids in cherry wine was found to be 72.78 mg L^−1^, which is much higher than in grape wines, where it varies from 28.63 mg L^−1^ in Cabernet Sauvignon-North Serbia, to 48.90 mg L^−1^ in Vranac.

The following findings further highlight characteristic profile of cherry wine: naringenin and apigenin were found only in cherry wine (0.15 mg L^−1^ and 0.06 mg L^−1^); ellagic acid, myricetin and naringin were not found in cherry wine, but they were found in all grape wine samples. The gallic acid was the major phenolic compound in grape wines (12.56–30.39 mg L^−1^), while its content was up to 30 times lower in cherry wine.

From the qualitative profile obtained from the UHPLC-MS/MS Orbitrap analysis (Table 3) one can conclude that isomers of caffeoylquinic acids, quercetin-3-O-hexosides, and caffeoyl-hexosides were found only in cherry wine. Coumaroyl-hexosides,* trans-*caftaric acid, dihydroquercetin-3-O-rhamnoside, procyanidin B type isomer 3, myricetin-3-O-hexoside, dihydromyricetin-3-O-rhamnoside, and luteolin, were found only in grape wine samples.

**Figure 2:**
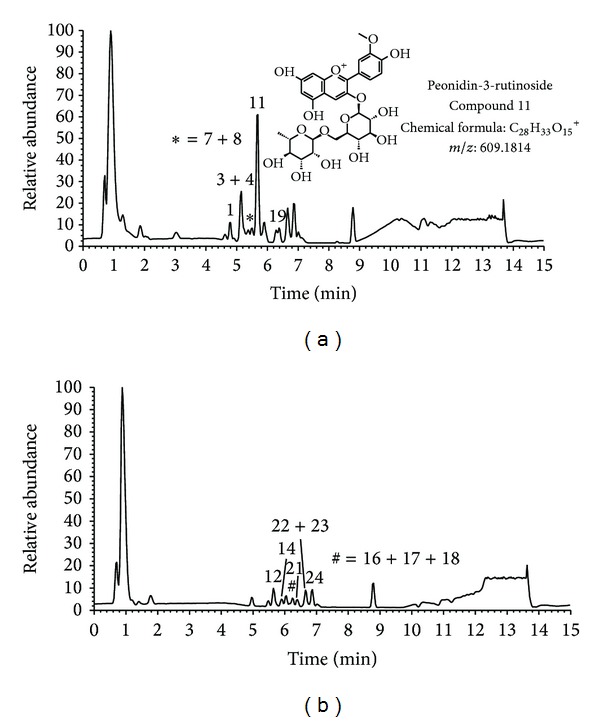
Base peak chromatograms of (a) cherry wine and (b) Cabernet Sauvignon-Central Serbia in positive ion mode.

**Figure 3:**
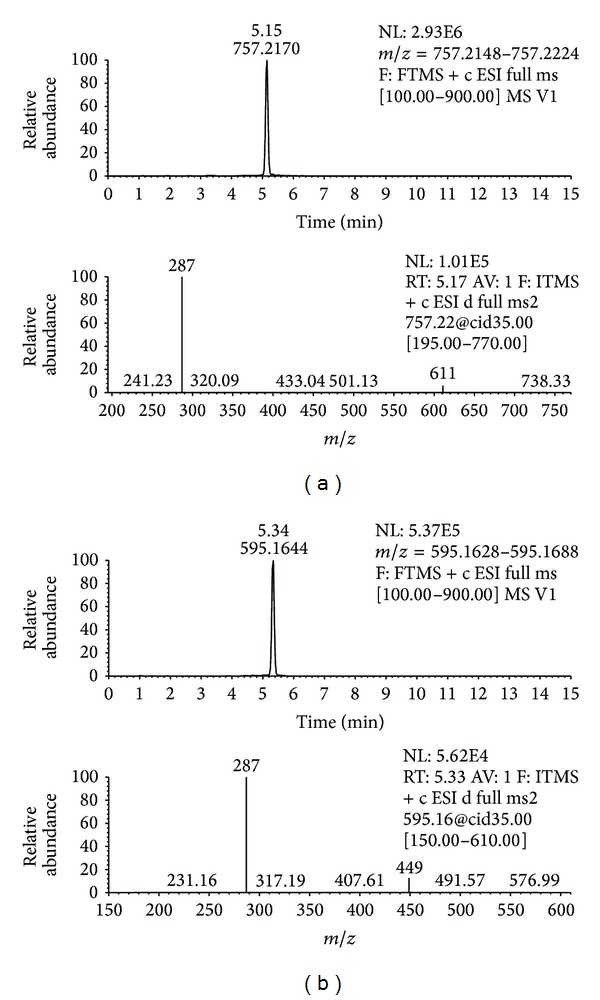
Extracted ion chromatograms and MS/MS spectra of (a) cyanidin-3-glucosylrutinoside and (b) cyanidin-3-rutinoside.

A total of 24 anthocyanin derivatives of were identified based on the search for the M^+^ molecular ion together with the interpretation of its fragmentations (Table 4). Anthocyanin profile of cherry wine was shown to be distinctive when compared to grape wines. Differences are obvious from the visual inspection of the two base peak chromatograms obtained in positive ion mode. Only as an example, in Figure 2 two base peak chromatograms, for cherry wine (a) and grape wine Cabernet Sauvignon-Central Serbia (b) are presented. Extracted ion chromatograms in positive ion mode and MS/MS data of the most abundant anthocyanins found in sour cherry wine are presented in Figure 3: (a) cyanidin-3-glucosylrutinoside and (b) cyanidin-3-rutinoside. The only anthocyanin common to all tested samples was cyanidin-3-glucoside. As it was already stated in the introduction section, published literature indicates seven anthocyanins to occur in cherries. Apart from these seven, two more namely delphinidin-3-rutinoside and pelargonidin-3-glucosylrutinoside were identified in this study. Other derivatives of delphinidin, malvidin, and petunidin were not found in cherry wine, while their presence was confirmed in grape wines, except for malvidin-3-caffeoylglucoside which was detected in two wines: samples W4 and W5. When comparing anthocyanins in all investigated wines, a total of seven compounds were distinctive for cherry wine (delphinidin-3-rutinoside, cyanidin-3-sophoroside, cyanidin-3-pentosylrutinoside, pelargonidin-3-glucosylrutinoside, cyanidin-3-rutinoside, peonidin-3-rutinoside, and pelargonidin-3-glucoside).

### 3.3. Determination of Minerals

The content of minerals is presented in Table 5. Most common element in all samples was potassium. Cherry wine contains up to five times higher amounts of this mineral than grape wines (content ranging from 246.200 mg kg^−1^, in Vranac, to 1373.100 mg kg^−1^ in cherry wine). Higher contents of P, Ca and Mg were found in cherry wine than in grape wine samples. Toxic elements (As, Cd and Pb) were found in small amounts in all tested wines (the allowable levels are 0.2 mg kg^−1^ for Pb and As, and 0.01 mg kg^−1^ for Cd) [19].

## 4. Conclusions

This paper was aimed at characterizing the wine obtained from a native cultivar of sour cherry Oblačinska. The significance of this work is primarily in chemical characterization and comprehensive data collection on the sour cherry wine produced from this cultivar. TPC, TAC and RSA values of cherry wine were in the range of the results determined for red wines. The results suggest that cherry wine contains a high concentration of different polyphenols with high antioxidant potential. Polyphenolic profile of cherry wine varied considerably when compared to grape wines. Caffeic acid, chlorogenic acid, protocatechuic acid, and p-coumaric acid were the main phenolic acids found in cherry wine, and the contents of these acids were much higher in cherry wine than in grape wines tested. As for flavonoids, naringenin and apigenin were found only in cherry wine. A total of seven anthocyanins were found in cherry wine only (delphinidin-3-rutinoside, cyanidin-3-sophoroside, cyanidin-3-pentosylrutinoside, pelargonidin-3-glucosylrutinoside, cyanidin-3-rutinoside, peonidin-3-rutinoside, and pelargonidin-3-glucoside).

## Figures and Tables

**Table 1:** Total phenolics contents, total anthocyanin contents, and radical scavenging activity in six wine samples^a^.

Sample	TPC (g GAE L^−1^)	TAC (g mal-3-glu L^−1^)	RSA(%)
SCW	1.94 ± 0.04	0.12 ± 0.01	34.56 ± 0.18
W1	1.76 ± 0.02	0.08 ± 0.01	32.06 ± 0.09
W2	2.28 ± 0.11	0.17 ± 0.02	46.41 ± 0.20
W3	2.50 ± 0.11	0.10 ± 0.01	48.60 ± 0.13
W4	1.19 ± 0.01	0.17 ± 0.02	21.00 ± 0.05
W5	1.69 ± 0.02	0.22 ± 0.02	31.21 ± 0.24

^a^The values shown are mean ± standard deviation of three replications.

**Table 2:** Contents of phenolics and *cis,trans*-abscisic acid (mg kg^−1^) in wine samples (ND = not detected compound). Results are expressed as mg L^−1^.

Compound	*t*_*R*_, min	SCW	W1	W2	W3	W4	W5
Gallic acid (**A**)^a^	1.79	1.10	28.57	20.73	30.39	12.56	20.86
Protocatechuic acid (**B**)	3.73	23.89	7.93	6.11	4.52	5.38	5.19
(−)-Gallocatechin (**B**)	3.78	ND	ND	4.62	6.02	5.47	2.59
Aesculin (**C**)	4.78	0.35	0.54	0.37	0.40	0.36	0.45
(−)-Epigallocatechin (**D**)	4.89	1.01	0.87	0.87	1.07	0.91	0.82
*p*-Hydroxybenzoic acid (**E**)	5.10	6.65	4.30	7.45	5.34	0.37	10.45
Gentisic acid (**E**)	5.12	0.27	0.16	0.42	0.24	1.00	0.14
Chlorogenic acid (**F**)	5.22	3.57	0.60	0.58	0.58	ND	0.58
(+)-Catechin (**G**)	5.30	1.31	3.54	5.86	6.13	5.21	14.09
Caffeic acid (**H**)	5.52	13.88	1.56	2.35	1.25	1.42	1.60
(−)-Epicatechin (**I**)	5.65	3.92	ND	3.26	3.20	1.86	6.86
(−)-Gallocatechin gallate (**J**)	5.79	ND	ND	ND	ND	ND	2.79
Rutin (**K**)	6.08	0.23	0.23	0.23	0.24	0.23	ND
*p*-Coumaric acid (**L**)	6.20	23.42	3.62	7.77	3.19	5.86	2.98
Ellagic acid (**L**)	6.24	ND	2.16	2.73	1.99	2.04	1.79
Naringin (**M**)	6.46	ND	0.31	0.90	0.90	0.79	0.97
(−)-Epigallocatechin gallate (**N**)	6.79	ND	2.58	0.90	2.36	1.03	ND
Myricetin (**O**)	6.93	ND	0.21	0.22	0.28	0.25	0.29
*cis, trans*-Abscisic acid (**P**)	7.43	1.06	0.17	0.30	0.21	0.11	0.10
Quercetin (**S**)	7.60	ND	ND	ND	0.03	ND	ND
Resveratrol (**S**)	7.65	ND	ND	ND	8.83	ND	ND
Naringenin (**T**)	8.06	0.15	ND	ND	ND	ND	ND
Apigenin (**U**)	8.20	0.06	ND	ND	ND	ND	ND
Hesperetin (**W**)	9.51	ND	0.27	ND	0.39	0.42	ND

^a^Corresponding to Figure 1.

**Table 3:** Characterization of phenolic compounds in wine samples using UHPLC − MS/MS Orbitrap. Target compounds, mean expected retention times (*t*_*R*_), calculated mass, found mass, mean mass accuracy (ppm), and MS/MS fragments (+ stands for detected and ND for not detected compound).

Compound	*t*_*R*_, min	Calculated [M–H]^−^	Found [M–H]^−^	ppm	MS/MS fragments	SCW	W1	W2	W3	W4	W5
Caffeoylquinic acid isomer 1	2.52	353.0867	353.0855	3.4	179, 191	+	ND	ND	ND	ND	ND
*trans*-Caftaric acid	2.54	311.0407	311.0393	4.5	135, 149, 179	ND	+	+	+	+	+
Quercetin-3-*O*-hexoside 1	2.56	463.0882	463.0859	5.0	179, 301	+	ND	ND	ND	ND	ND
Quercetin-3-*O*-hexoside 2	2.99	463.0882	463.0860	4.8	179, 301	+	ND	ND	ND	ND	ND
Procyanidin B type isomer 1	3.14	577.1351	577.1325	4.5	425	+	+	+	+	+	+
Caffeoyl-hexoside 1	3.52	341.0874	341.0869	1.5	179, 135	+	ND	ND	ND	ND	ND
Caffeoyl-hexoside 2	3.60	341.0874	341.0874	0.0	179, 135	+	ND	ND	ND	ND	ND
Caffeoylquinic acid isomer 2	3.61	353.0867	353.0856	3.1	179, 191	+	ND	ND	ND	ND	ND
Coumaroyl-hexoside 1	3.64	325.0929	325.0913	4.9	163	ND	+	ND	+	+	+
Procyanidin B type isomer 2	3.69	577.1351	577.1325	4.5	425	+	+	+	+	+	+
Coumaroyl-hexoside 2	3.72	325.0929	325.0919	3.1	163	ND	+	+	+	+	+
Coumaroyl-hexoside 3	3.89	325.0929	325.0913	4.9	163	ND	+	+	+	+	+
Dihydroquercetin-3-*O*-rhamnoside	3.97	449.1074	449.1064	2.2	303	ND	+	+	+	+	+
Procyanidin B type isomer 3	4.09	577.1351	577.1325	4.5	425	ND	+	+	+	+	+
Myricetin-3-*O*-hexoside	4.14	479.0831	479.0810	4.4	317	ND	+	+	+	+	+
Dihydromyricetin-3-*O*-rhamnoside	4.22	465.1023	465.1015	1.7	319	ND	+	+	+	+	ND
Syringic acid	4.44	197.0455	197.0447	4.1	153	+	+	+	+	+	+
Cinnamic acid	4.56	147.0452	147.0448	2.7	103	ND	ND	+	+	+	ND
Luteolin^a^	5.55	285.0405	285.0393	4.2	133, 213	ND	+	+	+	+	+
Chrysin^a^	7.22	253.0506	253.0497	3.6	151, 181	+	+	+	+	+	ND
Pinocembrin^a^	7.34	255.0663	255.0651	4.7	213	+	+	ND	ND	+	+
Galangin^a^	7.34	269.0455	269.0443	4.5	183, 197	ND	+	+	+	+	ND

^a^Confirmed using available standards.

**Table 4:** Characterization of anthocyanins in wine samples using UHPLC + MS/MS Orbitrap. Target compounds, mean expected retention times (*t*_*R*_), calculated mass, found mass, mean mass accuracy (ppm), and MS/MS fragments (+ stands for detected and ND for not detected compound).

Peak number^a^	Compound	*t*_*R*_, min	Calculated M^+^	Found M^+^	ppm	MS/MS fragments	SCW	W1	W2	W3	W4	W5
1	Delphinidin-3-rutinoside	4.77	611.1598	611.1598	0.0	303, 449	+	ND	ND	ND	ND	ND
2	Delphinidin-3-glucoside	5.10	465.1023	465.1021	0.4	303	ND	+	+	+	+	+
3	Cyanidin-3-sophoroside	5.12	611.1598	611.1597	0.2	287, 449	+	ND	ND	ND	ND	ND
4	Cyanidin-3-glucosylrutinoside	5.15	757.2191	757.2170	2.8	287, 449, 611	+	+	ND	ND	ND	+
5	Cyanidin-3-pentosylrutinoside	5.25	727.2072	727.2069	0.4	287, 449, 581	+	ND	ND	ND	ND	ND
6	Cyanidin-3-glucoside	5.30	449.1074	449.1072	0.4	287	+	+	+	+	+	+
7	Pelargonidin-3-glucosylrutinoside	5.31	741.2228	741.2225	0.4	271, 433, 579	+	ND	ND	ND	ND	ND
8	Cyanidin-3-rutinoside	5.34	595.1653	595.1644	1.5	287, 449	+	ND	ND	ND	ND	ND
9	Petunidin-3-glucoside	5.38	479.1180	479.1186	−1.3	317	ND	+	+	+	+	+
10	Peonidin-3-glucoside	5.58	463.1231	463.1228	0.6	301	ND	+	+	+	+	+
11	Peonidin-3-rutinoside	5.60	609.1810	609.1808	0.3	301, 463	+	ND	ND	ND	ND	ND
12	Malvidin-3-glucoside	5.65	493.1336	493.1334	0.4	331	ND	+	+	+	+	+
13	Delphinidin-3-acetylglucoside	5.71	507.1123	507.1125	−0.4	303	ND	+	+	+	+	+
14	Cyanidin-3-acetylglucoside	5.93	491.1174	491.1174	0.0	287	ND	+	+	+	+	+
15	Petunidin-3-acetylglucoside	5.99	521.1280	521.1281	−0.2	317	ND	+	+	+	+	+
16	Cyanidin-3-pentoside	6.20	419.0968	419.0966	0.5	287	ND	+	+	+	+	+
17	Peonidin-3-acetylglucoside	6.21	505.1331	505.1334	−0.6	301	ND	+	+	+	+	+
18	Malvidin-3-acetylglucoside	6.24	535.1436	535.1437	−0.2	331	ND	+	+	+	+	+
19	Pelargonidin-3-glucoside	6.31	433.1125	433.1125	0.0	271	+	ND	ND	ND	ND	ND
20	Malvidin-3-caffeoylglucoside	6.39	655.1649	655.1643	0.9	331	ND	ND	ND	ND	+	+
21	Petunidin-3-*cis*-*p*-coumaroylglucoside	6.43	625.1543	625.1547	−0.6	317	ND	+	+	+	+	+
22	Malvidin-3-*cis*-*p*-coumaroylglucoside	6.68	639.1700	639.1696	0.6	331	ND	+	+	+	+	+
23	Petunidin-3-*trans*-*p*-coumaroylglucoside	6.68	625.1543	625.1547	−0.6	317	ND	+	+	+	+	+
24	Malvidin-3-*trans*-*p*-coumaroylglucoside	6.93	639.1700	639.1696	0.6	331	ND	+	+	+	+	+

^a^Peak numbers correspond to Figure 2.

**Table 5:** The amounts of minerals in cherry wine and grape wine samples.

Mineral	SCW	W1	W2	W3	W4	W5
Al (mg kg^−1^)	0.200	0.070	0.071	0.421	0.055	0.090
As (*μ*g kg^−1^)	0.093	0.060	0.063	0.108	0.042	0.073
B (mg kg^−1^)	2.760	1.364	2.101	1.919	1.690	2.082
Ca (g kg^−1^)	0.084	0.035	0.023	0.041	0.020	0.022
Cd (*μ*g kg^−1^)	0.093	0.002	0.063	0.051	0.042	0.073
Co (*μ*g kg^−1^)	0.577	0.755	0.313	0.501	0.143	0.512
Cr (mg kg^−1^)	0.016	0.006	0.004	0.005	0.002	0.004
Cu (mg kg^−1^)	0.030	0.015	0.041	0.143	0.016	0.041
Fe (mg kg^−1^)	2.192	0.575	0.221	2.415	0.642	1.114
K (g kg^−1^)	1.373	0.246	0.521	0.499	0.283	0.475
Li (*μ*g kg^−1^)	0.678	1.916	2.726	2.794	2.162	1.215
Mg (g kg^−1^)	0.072	0.052	0.052	0.065	0.041	0.054
Mn (mg kg^−1^)	0.632	0.377	0.532	0.489	0.783	0.470
Mo (*μ*g kg^−1^)	0.093	0.060	0.063	0.108	0.042	0.073
Na (mg kg^−1^)	1.650	2.228	2.814	4.227	0.893	4.507
Ni (mg kg^−1^)	0.054	0.027	0.018	0.019	0.007	0.012
P (g kg^−1^)	0.179	0.113	0.137	0.109	0.080	0.129
Pb (*μ*g kg^−1^)	4.404	3.757	0.063	18.490	0.380	28.868
S (g kg^−1^)	0.089	0.093	0.144	0.169	0.066	0.118
Sb (*μ*g kg^−1^)	0.093	0.060	0.063	1.670	0.042	0.073
Se (mg kg^−1^)	0.011	0.009	0.016	0.009	0.012	0.008
V (*μ*g kg^−1^)	6.863	2.515	0.323	0.947	0.498	2.237
Zn (mg kg^−1^)	0.311	0.131	0.142	0.327	0.059	0.156
